# *ν*-Support Vector Regression Model Based on Gauss-Laplace Mixture Noise Characteristic for Wind Speed Prediction

**DOI:** 10.3390/e21111056

**Published:** 2019-10-28

**Authors:** Shiguang Zhang, Ting Zhou, Lin Sun, Wei Wang, Chuan Wang, Wentao Mao

**Affiliations:** 1 College of Computer and Information Engineering, Henan Normal University, Xinxiang 453007, China; wangwei@htu.edu.cn (W.W.);; 2 School of Computer Science and Technology, Tianjin University, Tianjin 300350, China; 3 Engineering Lab of Intelligence Business and Internet of Things, Xinxiang 453007, China; 4 The State-Owned Assets Management Office, Henan Normal University, Xinxiang 453007, China

**Keywords:** *ν*-support vector regression, Gauss-Laplace mixture noise, empirical risk loss, inequality constraints, wind speed prediction

## Abstract

Most regression techniques assume that the noise characteristics are subject to single noise distribution whereas the wind speed prediction is difficult to model by the single noise distribution because the noise of wind speed is complicated due to its intermittency and random fluctuations. Therefore, we will present the ν-support vector regression model of Gauss-Laplace mixture heteroscedastic noise (GLM-SVR) and Gauss-Laplace mixture homoscedastic noise (GLMH-SVR) for complex noise. The augmented Lagrange multiplier method is introduced to solve models GLM-SVR and GLMH-SVR. The proposed model is applied to short-term wind speed forecasting using historical data to predict future wind speed at a certain time. The experimental results show that the proposed technique outperforms the single noise technique and obtains good performance.

## 1. Introduction

Wind speed and wind power prediction is becoming increasingly important, and wind speed prediction is crucial for the control, scheduling, maintenance, and resource planning of wind energy conversion systems [1,2]. However, the volatility and uncertainty of wind speed give a fundamental challenge to power system operations. Because the basic characteristics of the wind is its intermittency and random fluctuations [3,4], the integration of wind power into power systems puts forward a series of challenges. The most effective way to resolve the challenges is to improve the prediction accuracy of wind speed and power forecasting [5,6,7].

In general, there are three important types in building a regression algorithm: model structures, objective functions, and optimization strategies. The model structures include linear or nonlinear functions, neural networks [8,9,10], etc. As for objective functions, empirical risk loss has a great effect on the performance of regression models. The selection of empirical risk loss is mostly dependent on the types of noises [11,12]. For example, squared loss is suitable for Gaussian noise [13,14,15], least absolute deviation loss for Laplacian noise [16], and Beta loss for Beta noise [17,18,19]. By the formula of the optimization method, a series of optimization algorithms are developed [20]. This work mainly studies what should be considered in the optimal architecture of the support vector regression (SVR) model in complex or unknown noise.

Recently, SVR has become an increasingly important technology. In 2000, ν-SVR was introduced by Schölkopf, et al. [21] and automatically computes ϵ. Suykens et al. [22,23] constructed least squares SVR with Gaussian noise (LS-SVR). Wu [13] and Pontil et al. [24] constructed ν-SVR with Gaussian noise (GN-SVR). In 2002, Bofinger et al. [25] discovered that the output of a wind turbine system is limited between zero and maximum power and that the error statistics do not follow a normal distribution. In 2007, Zhang et al. [26] and Randazzo et al. [27] proposed the estimation of coherent electromagnetic wave impact in the direction of arrival under Laplace noise environment. Bludszuweit et al. [28] explained the advantages of using Beta probability density function (PDF) instead of Gauss PDF to approximate the error distribution of wind power forecasting. According to Bayesian principle, square loss, Beta loss, or Laplacian loss are optimal when the noise is Gaussian, Beta, or Laplacian, respectively [17,18]. However, in some real-world applications, the noise distribution is complex and unknown if the data are collected in muti-source environments. Therefore, a single distribution attended to describes clearly that the real noise is not optimal and almost impossible [29,30]. Generally speaking, mixture distributions have good approximation capability for any continuous distributions. It can adapt well to unknown or complex noises when we have no prior knowledge of real noise. In 2017, the hybrid forecasting model based on multi-objective optimization [29,31], a hybrid method based on singular spectrum analysis, firefly algorithm, and BP neural network forecast the wind speed of complex noise [32]; this shows that the hybrid method has strong prediction ability. The hybrid of least squares support vector machine [33] is applied to predict the wind speed of unknown noise, which improves the forecasting performance of wind speed. Two novel nonlinear regression models [34] where the noise is fitted by mixture of Gaussian were developed, produced good performance compared with current regression algorithms, and provided superior robustness.

To address the above problem, we try to study the ν-SVR model of Gauss-Laplace mixture noise characteristics for complex or unknown noise distribution. In this case, we must design a method to find the optimal solution of the corresponding regression task. Although there has been a large number of SVR algorithm implementations in the past few years, we introduced the augmented Lagrange multiplier method (ALM) method described in Section 4. Sub-gradient descent method can be used if the task is non-differentiable or discontinuous [17], or sequence minimum optimization algorithm (SMO) can be used if the sample size is large [35].

This work offers the following four contributions: (1) the optimal empirical risk loss for general mixture noise characteristic and Gauss-Laplace mixture noise by the use of Bayesian principle is obtained; (2) the ν-SVR model of mixture noise, Gauss-Laplace mixture homoscedastic noise (GLM-SVR), and Gauss-Laplace mixture heteroscedastic noise (GLMH-SVR) for complex or unknown noise is constructed; (3) the augmented Lagrange multiplier method is applied to solve GLM-SVR, which guarantees the stability and validity of the solution; and (4) GLM-SVR is applied to short-term wind speed forecasting using historical data to predict future wind speed at a certain time and to verify the validity of the proposed technique.

A summary of the rest of this article is organized as follows. Section 2 derives the optimal empirical risk loss using Bayesian principle; Section 3 constructs the ν-SVR model of Gauss-Laplace mixture noise characteristics; Section 4 gives the solution and algorithm design of GLM-SVR; numerical experiments are conducted out on short-term wind speed prediction in Section 5; and Section 6 summarizes this article.

## 2. Bayesian Principle to Empirical Risk Loss of Mixture Noise

In this section, using the theory of Bayesian principle, we obtain the optimal empirical risk loss of mixture noise characteristics.

Given the following dataset

(1) $$D_{L}=\left\{\left(X_{1},Y_{1}\right),\left(X_{2},Y_{2}\right),\cdots ,\left(X_{L},Y_{L}\right)\right\},$$

where $X_{i}={\left(x_{i1},x_{i2},\cdots ,x_{in}\right)}^{T}\in R^{n}$ and $Y_{i}\in R\left(i=1,2,\cdots ,L\right)$ are the datasets, *R* represents real number set, $R^{n}$ is the *n* dimensional Euclidean space, *L* is the number of sample points, and superscript *T* denotes the matrix transpose, suppose the sample of dataset $D_{L}$ is generated by the additive noise function ε; the following relationship between the measured values $Y_{i}$ and predicted values $f(X_{i})$ is as follows:

(2) $$Y_{i}=f\left(X_{i}\right)+\varepsilon_{i}\left(i=1,2,\cdots ,L\right)$$

where $\varepsilon_{i}$ be random and i.i.d. means independent and identical distribution with $P(\varepsilon_{i})$ of mean μ and standard deviation σ. In engineering technology, the noise density P(ε)=P(Y−f(X)) is unknown. We want to predict the unknown decision function f(X) from the training samples $D_{f}\subseteq D_{L}$.

Following References [24,36] by the use of Bayesian principle, in maximum likelihood sense, the optimal empirical risk loss is as follows:

(3) l(ε)=l(X,Y,f(X))=−logP(Y−f(X)).

i.e., the optimal empirical risk loss l(ε) is the log-likelihood of the noise model.

The probability density function (PDF) of each single distribution model and the parameters estimation formula under Bayesian principle are summarized in Reference [16]. In particular, the noise ε in Equation (2) is Laplacian, with PDF $P\left(\varepsilon \right)=\frac{1}{2}e^{-|\varepsilon |}$. By Equation (3), the optimal empirical risk loss in the sense of maximum likelihood sense should be l(ε)=|ε|. If the noise in Equation (2) is Gaussian, with zero mean and homoscedastic standard deviation σ, by Equation (3), empirical risk loss about Gaussian noise is $l\left(\varepsilon \right)=\frac{1}{2\sigma^{2}}\cdot \varepsilon^{2}$. Suppose the noise ε in Equation (2) is Gaussian, with zero mean and heteroscedastic standard deviation $\sigma_{i}$(i=1,2,⋯,L). By Equation (3), the loss about Gaussian noise is $l\left(\varepsilon_{i}\right)=\frac{1}{2\sigma_{i}^{2}}\cdot \varepsilon_{i}^{2}$ (i=1,⋯,L).

It is assumed that the noise ε in Equation (2) is the mixture distributions of two kinds of noise characteristics with the probability density functions $P_{1}\left(\varepsilon \right)$ and $P_{2}\left(\varepsilon \right)$, respectively. Suppose that $P\left(\varepsilon \right)=\left[P_{1}{\left(\varepsilon \right)}^{\lambda_{1}}\right]\cdot \left[P_{2}{\left(\varepsilon \right)}^{\lambda_{2}}\right]$, by Equation (3), the optimal empirical risk loss about the mixture noise distributions is as follows:

(4) $$l\left(\varepsilon \right)=\lambda_{1}\cdot l_{1}\left(\varepsilon \right)+\lambda_{2}\cdot l_{2}\left(\varepsilon \right).$$

where $l_{1}\left(\varepsilon \right)>0,l_{2}\left(\varepsilon \right)>0$ are convex empirical risk losses of the above two kinds of noise characteristics, respectively. Weight factor $\lambda_{1},\lambda_{2}\geq 0$ and $\lambda_{1}+\lambda_{2}=1$.

**Figure 1:**
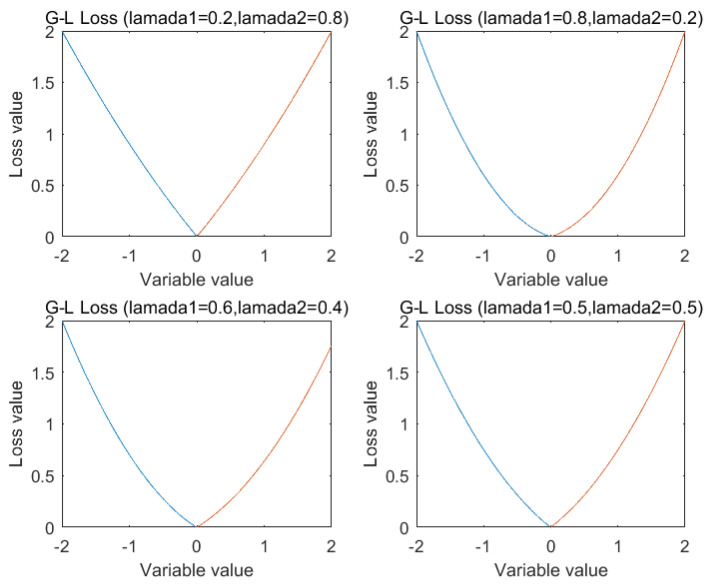
Gauss-Laplace empirical risk loss for different parameter.

The Gauss-Laplace empirical risk loss for different parameter are shown in Figure 1.

## 3. Model ν-SVR of Gauss-Laplace Mixture Noise

Given dataset $D_{L}$, we build a linear regressor $f\left(X\right)=\varpi^{T}\cdot X+b$, where ϖ denotes the weight vector and *b* is the bias term. To deal with nonlinear problems, the following summaries can be made [37,38]: the input vector $X_{i}\in R^{n}$ is mapped by a nonlinear mapping (chosen a priori) Φ: $R^{n}\to H$ is the high dimensional feature space *H* (*H* is Hilbert space), induced by the kernel matrix $K\left(X_{i},X_{j}\right)=\left(\Phi \left(X_{i}\right)\cdot \Phi \left(X_{j}\right)\right)$ (i,j=1,2,⋯,L). $\left(\Phi \left(X_{i}\right)\cdot \Phi \left(X_{j}\right)\right)$ is the inner product in *H*, and the kernel mapping Φ may be any positive definite Mercer kernel. Therefore, we will solve the optimization problem in feature space *H*. The linear ν-SVR is extended to the nonlinear ν-SVR by using the kernel matrix $K(X_{i},X_{j})$.

We propose the uniform model ν-SVR of mixture noises (M-SVR). The primal problem of model M-SVR is described as follows:

(5) $$\begin{array}{c} Min\{g_{P_{M-SVR}}=\frac{1}{2}\varpi^{T}\cdot \varpi +\frac{C}{L}\cdot [\nu \cdot \varepsilon +\lambda_{1}\cdot \sum_{i=1}^{L}\left(l_{1}\left(\xi_{i}\right)+l_{1}\left(\xi_{i}^{*}\right)\right)\\ \;\;\;\;\;+\lambda_{2}\cdot \sum_{i=1}^{L}\left(l_{2}\left(\xi_{i}\right)+l_{2}\left(\xi_{i}^{*}\right)\right)]\}\\ Subject\;to:\varpi^{T}\cdot \Phi \left(X_{i}\right)+b-Y_{i}\leq \varepsilon +\xi_{i}\\ \;\;\;\;\;\;Y_{i}-\varpi^{T}\cdot \Phi \left(X_{i}\right)-b\leq \varepsilon +\xi_{i}^{*} \end{array}$$

where $\xi_{i}$ and $\xi_{i}^{*}$ are random noises and slack variable at time *i*. $l_{1}\left(\xi_{i}\right),l_{1}\left(\xi_{i}^{*}\right),l_{2}\left(\xi_{i}\right)$, and $l_{2}\left(\xi_{i}^{*}\right)>0$ (i=1,2,⋯,L) are convex empirical risk loss function values for general noise characteristic in the sample point $\left(X_{i},Y_{i}\right)\in D_{L}$ (i,j=1,2,⋯,L). C>0 is the penalty parameter, ε≥0, and ν∈(0,1]. Weight factor $\lambda_{1},\lambda_{2}\geq 0$ and $\lambda_{1}+\lambda_{2}=1$.

As a function approximation machine, the objection is to estimate an unknown function f(X) from the training samples $D_{f}\subseteq D_{L}$. In the field of practical application, most of the distributions do not satisfy the Gauss distribution, and it also does not obey the Laplace distribution. The noise distribution is unknown or complex; a single distribution intended to describe the real noise is almost impossible. Generally, mixture distributions (as Gauss-Laplace mixed distribution) have good approximation capabilities for any continuous distributions, and it can fit the unknown or complex noise. Therefore, we will use the Gauss-Laplace mixed homoscedastic and heteroscedastic noise distribution to fit the unknown or complex noise characteristics in the next section.

### 3.1. Model ν-SVR of Gauss-Laplace Mixture Homoscedastic Noise

If the noise in Equation (2) is Gaussian, with zero mean and the homoscedastic standard deviation σ, by Equation (3), the empirical risk loss of homoscedastic Gaussian noise is $l_{1}\left(\xi_{i},\xi_{i}^{*}\right)=\frac{1}{2\sigma^{2}}\left(\xi_{i}^{2}+{\left(\xi_{i}^{*}\right)}^{2}\right)$ and the empirical risk loss of Laplace noise is $l_{2}\left(\xi_{i},\xi_{i}^{*}\right)=|\xi_{i}\left|+\right|\xi_{i}^{*}|$. We adopt the Gauss-Laplace mixture homoscedastic noise distribution to fit the unknown noise characteristics. By Equation (4), the loss function corresponding to Gauss-Laplace mixture homoscedastic noise characteristics is $l\left(\xi_{i},\xi_{i}^{*}\right)=\frac{\lambda_{1}}{2}\cdot \left(\xi_{i}^{2}+{\left(\xi_{i}^{*}\right)}^{2}\right)+\lambda_{2}\cdot \left(\right|\xi_{i}\left|+\right|\xi_{i}^{*}\left|\right)$. We put forward a technique of ν-SVR model for Gauss-Laplace mixture homoscedastic noise characteristics (GLM-SVR). The primal problem of model GLM-SVR be described as follows:

(6) $$\begin{array}{c} Min\{g_{P_{GLM-SVR}}=\frac{1}{2}\varpi^{T}\cdot \varpi +\frac{C}{L}\cdot [\nu \cdot \varepsilon +\frac{\lambda_{1}}{2}\cdot \sum_{i=1}^{L}\left(\xi_{i}^{2}+{\left(\xi_{i}^{*}\right)}^{2}\right)\\ \;\;\;\;\;+\lambda_{2}\cdot \sum_{i=1}^{L}\left(\xi_{i}+\xi_{i}^{*}\right)]\}\\ Subject\;to:\varpi^{T}\cdot \Phi \left(X_{i}\right)+b-Y_{i}\leq \varepsilon +\xi_{i}\\ \;\;\;\;\;\;Y_{i}-\varpi^{T}\cdot \Phi \left(X_{i}\right)-b\leq \varepsilon +\xi_{i}^{*} \end{array}$$

where $\xi_{i},\xi_{i}^{*}\geq 0\left(i=1,2,\cdots ,L\right)$ are random noises and slack variables at time *i*. C>0 is the penalty parameter, ε≥0, and ν∈(0,1]. Weight factor $\lambda_{1},\lambda_{2}\geq 0$ and $\lambda_{1}+\lambda_{2}=1$.

**Proposition** **1.** *The solution of the primal problem Equation (6) of model GLM-SVR about ϖ exists and is unique.*

**Theorem** **1.** *The dual problem of the primal problem of Equation (6) of model GLM-SVR is as follows:*

(7) $$\begin{array}{c} Max\{g_{D_{GLM-SVR}}=-\frac{1}{2}\sum_{i=1}^{L}\sum_{j=1}^{L}\left(\alpha_{i}^{*}-\alpha_{i}\right)\cdot \left(\alpha_{j}^{*}-\alpha_{j}\right)\cdot K\left(X_{i},X_{j}\right)\\ \;\;\;\;\;+\sum_{i=1}^{L}\left(\alpha_{i}^{*}-\alpha_{i}\right)\cdot Y_{i}-\frac{L}{2C\cdot \lambda_{1}}\sum_{i=1}^{L}[{\left(\alpha_{i}-C\cdot \lambda_{2}\right)}^{2}\\ \;\;\;\;\;+{\left(\alpha_{i}^{*}-C\cdot \lambda_{2}\right)}^{2}]\}\\ Subject\;to\;\sum_{i=1}^{L}\left(\alpha_{i}^{*}-\alpha_{i}\right)=0\\ \;\;\;\;\;\;0\leq \alpha_{i},\alpha_{i}^{*}\leq \frac{C}{L},i=1,2,\cdots ,L\\ \;\;\;\;\;\;\sum_{i=1}^{L}\left(\alpha_{i}^{*}+\alpha_{i}\right)\leq C\cdot \nu . \end{array}$$

where $\alpha_{i},\alpha_{i}^{*}\left(i=1,2,\cdots ,L\right)$ are Lagrange multipliers and $K(X_{i},X_{j})$(i,j=1,2,⋯,L) is a kernel matrix. C>0 is the penalty parameter, ν∈(0,1]. Weight factor $\lambda_{1},\lambda_{2}\geq 0$ and $\lambda_{1}+\lambda_{2}=1$.

**Proof.**  Let us take Lagrange functional $L_{\varpi }\left(\varpi ,b,\alpha ,\alpha^{*},\xi ,\xi^{*},\eta ,\eta^{*},\varepsilon ,\gamma \right)$ as

$$\begin{array}{c} L_{\varpi }=\frac{1}{2}\varpi^{T}\cdot \varpi +\frac{C}{L}\cdot \left[\nu \cdot \varepsilon +\frac{\lambda_{1}}{2}\cdot \sum_{i=1}^{L}\left(\xi_{i}^{2}+{\left(\xi_{i}^{*}\right)}^{2}\right)+\lambda_{2}\cdot \sum_{i=1}^{L}\left(\xi_{i}+\xi_{i}^{*}\right)\right]-\gamma \varepsilon \\ -\sum_{i=1}^{L}\left(\eta_{i}\xi_{i}+\eta_{i}^{*}\xi_{i}^{*}\right)-\sum_{i=1}^{L}\alpha_{i}\left(\xi_{i}+Y_{i}-\varpi^{T}\cdot \Phi \left(X_{i}\right)-b+\varepsilon \right)\\ -\sum_{i=1}^{L}\alpha_{i}^{*}\left(\xi_{i}^{*}-Y_{i}+\varpi^{T}\cdot \Phi \left(X_{i}\right)+b+\varepsilon \right). \end{array}$$

To minimize $L_{\varpi }$, let us find partial derivatives $\varpi ,b,\xi ,\xi^{*},$ and ε. On the basis of KKT (Karush–Kuhn–Tucker) conditions, we get

$$\nabla_{\varpi }\left(L_{\varpi }\right)=0,\nabla_{b}\left(L_{\varpi }\right)=0,\nabla_{\varepsilon }\left(L_{\varpi }\right)=0,\nabla_{\xi }\left(L_{\varpi }\right)=0,\nabla_{\xi^{*}}\left(L_{\varpi }\right)=0.$$

and have

$$\varpi_{i}=\sum_{i=1}^{L}\left(\alpha_{i}^{*}-\alpha_{i}\right)\cdot \Phi \left(X_{i}\right),$$

$$\sum_{i=1}^{L}\left(\alpha_{i}^{*}-\alpha_{i}\right)=0,$$

$$\frac{C}{L}\cdot \nu -\gamma -\sum_{i=1}^{L}\left(\alpha_{i}^{*}+\alpha_{i}\right)=0,$$

$$\frac{C}{L}\cdot \left(\lambda_{1}\cdot \xi_{i}+\lambda_{2}\right)-\eta_{i}-\alpha_{i}=0,$$

$$\frac{C}{L}\cdot \left(\lambda_{1}\cdot \xi_{i}^{*}+\lambda_{2}\right)-\eta_{i}^{*}-\alpha_{i}^{*}=0,$$

By substituting the above extreme conditions into $L_{\varpi }$ and by seeking maximum $\alpha ,\alpha^{*}$, we obtain the dual problem of Equation (7) of the primal problem of Equation (6) of model GLM-SVR. □

Then, we obtain

$$\varpi_{i}=\sum_{i=1}^{L}\left(\alpha_{i}^{*}-\alpha_{i}\right)\cdot \Phi \left(X_{i}\right),$$

$$b=\frac{1}{L}\sum_{i=1}^{L}\left[Y_{i}-\sum_{i\in RSV}\left(\alpha_{i}^{*}-\alpha_{i}\right)\cdot K\left(X_{i},X_{j}\right)-\frac{\left(L\cdot \alpha_{i}-C\cdot \lambda_{2}\right)}{C\cdot \lambda_{1}}\right].$$

To estimate ε, we have

$$\varepsilon =\frac{1}{L}\sum_{j=1}^{L}\left(\sum_{i\in RSV}\left(\alpha_{i}^{*}-\alpha_{i}\right)\cdot K\left(X_{i},X_{j}\right)+b-Y_{j}\right).$$

Thus, the decision function of model GLM-SVR can be written as

$$f\left(X\right)=\varpi^{T}\cdot \Phi \left(X\right)+b=\sum_{i\in RSV}\left(\alpha_{i}^{*}-\alpha_{i}\right)K\left(X_{i},X\right)+b,$$

where RSVs are samples about $\alpha_{i}^{*}-\alpha_{i}\neq 0$ (called support vectors), $K\left(X_{i},X_{j}\right)=\left(\Phi \left(X_{i}\right)\cdot \Phi \left(X_{j}\right)\right)\left(i,j=1,2,\cdots ,L\right)$ is the Kernel function, $\Phi :R^{n}\to H$ ($R^{n}$ is the *n* dimensional Euclidean space, and *H* is the Hilbert space), and $\left(\Phi \left(X_{i}\right)\cdot \Phi \left(X_{j}\right)\right)$ is the inner product of *H*.

### 3.2. Model ν-SVR of Gauss-Laplace Mixture Heteroscedastic Noise

If the noise in Equation (2) is Gaussian, with zero mean and the heteroscedastic variance $\sigma_{i}^{2},{\left(\sigma_{i}^{*}\right)}^{2}$, that is $\sigma_{i}\neq \sigma_{j}$, $\sigma_{i}^{*}\neq \sigma_{j}^{*}$, where i≠j(i,j=1,⋯,L), by Equation (3), the empirical risk loss of heteroscedastic Gaussian noise characteristic is $l_{1}\left(\xi_{i},\xi_{i}^{*}\right)=\frac{1}{2\sigma_{i}^{2}}\xi_{i}^{2}+\frac{1}{2{\left(\sigma_{i}^{*}\right)}^{2}}{\left(\xi_{i}^{*}\right)}^{2}$, (i=1,⋯,L) and the empirical risk loss of Laplace noise characteristic is $l_{2}\left(\xi_{i},\xi_{i}^{*}\right)=|\xi_{i}\left|+\right|\xi_{i}^{*}|$. We utilize the Gauss-Laplace mixture heteroscedastic noise distribution to predict the unknown noise characteristics. By Equation (4), the empirical risk loss about Gauss-Laplace mixture heteroscedastic noise is $l\left(\xi_{i},\xi_{i}^{*}\right)=\frac{\lambda_{1}}{2}\cdot \left(\frac{1}{\sigma_{i}^{2}}\xi_{i}^{2}++\frac{1}{{\left(\sigma_{i}^{*}\right)}^{2}}{\left(\xi_{i}^{*}\right)}^{2}\right)+\lambda_{2}\cdot \left(\right|\xi_{i}\left|+\right|\xi_{i}^{*}\left|\right)$ (i=1,⋯,L). We propose a novel technique of ν-SVR model for Gauss-Laplace mixture heteroscedastic noise characteristics (GLMH-SVR). The primal problem of model GLMH-SVR can be formulated as follows:

(8) $$\begin{array}{c} Min\{g_{P_{GLMH-SVR}}=\frac{1}{2}\varpi^{T}\cdot \varpi +\frac{C}{L}\cdot [\nu \cdot \varepsilon +\frac{\lambda_{1}}{2}\cdot \sum_{i=1}^{L}(\frac{1}{\sigma_{i}^{2}}\cdot \xi_{i}^{2}+\\ \;\;\;\;\;\;\;\frac{1}{{\left(\sigma_{i}^{*}\right)}^{2}}\cdot {\left(\xi_{i}^{*}\right)}^{2})+\lambda_{2}\cdot \sum_{i=1}^{L}\left(\xi_{i}+\xi_{i}^{*}\right)]\}\\ Subject\;to:\varpi^{T}\cdot \Phi \left(X_{i}\right)+b-Y_{i}\leq \varepsilon +\xi_{i}\\ \;\;\;\;\;\;Y_{i}-\varpi^{T}\cdot \Phi \left(X_{i}\right)-b\leq \varepsilon +\xi_{i}^{*}. \end{array}$$

where $\xi_{i},\xi_{i}^{*}\geq 0,i=1,2,\cdots ,L$ are random noises and slack variables at time *i*, the variance $\sigma_{i}^{2},{\left(\sigma_{i}^{*}\right)}^{2}\left(i=1,2,\cdots ,L\right)$ is heteroscedastic, C>0 is the penalty parameter, ε≥0, and ν∈(0,1]. Weight factor $\lambda_{1},\lambda_{2}\geq 0$ and $\lambda_{1}+\lambda_{2}=1$.

**Proposition** **2.** *The solution of the primal problem of Equation (8) of GLMH-SVR about ω exists and is unique.*

**Theorem** **2.** *The dual problem of GLMH-SVR in the primal problem of Equation (8) is as follows:*

(9) $$\begin{array}{c} Max\{g_{D_{GLMH-SVR}}=-\frac{1}{2}\sum_{i=1}^{L}\sum_{j=1}^{L}\left(\alpha_{i}^{*}-\alpha_{i}\right)\cdot \left(\alpha_{j}^{*}-\alpha_{j}\right)\cdot K\left(X_{i},X_{j}\right)\\ +\sum_{i=1}^{L}\left(\alpha_{i}^{*}-\alpha_{i}\right)\cdot Y_{i}-\frac{L}{2C\cdot \lambda_{1}}[\sum_{i=1}^{L}{\left(\sigma_{i}^{2}\cdot \alpha_{i}-C\cdot \lambda_{2}\right)}^{2}\\ \;\;\;\;\;+\sum_{i=1}^{L}{\left({\left(\sigma_{i}^{*}\right)}^{2}\cdot \alpha_{i}^{*}-C\cdot \lambda_{2}\right)}^{2}]\}\\ Subject\;to\;\sum_{i=1}^{L}\left(\alpha_{i}^{*}-\alpha_{i}\right)=0\\ \;\;\;\;\;\;0\leq \alpha_{i}\leq \frac{C}{L\cdot \sigma_{i}^{2}},i=1,2,\cdots ,L\\ \;\;\;\;\;\;0\leq \alpha_{i}^{*}\leq \frac{C}{L\cdot {\left(\sigma_{i}^{*}\right)}^{2}},i=1,2,\cdots ,L\\ \;\;\;\;\;\;\sum_{i=1}^{L}\left(\alpha_{i}^{*}+\alpha_{i}\right)\leq C\cdot \nu ,i=1,2,\cdots ,L \end{array}$$

where $\sigma_{i}^{2},{\left(\sigma_{i}^{*}\right)}^{2}\left(i=1,2,\cdots ,L\right)$ is heteroscedastic, C>0 is the penalty parameter, and ν∈(0,1]. Weight factor $\lambda_{1},\lambda_{2}\geq 0$ and $\lambda_{1}+\lambda_{2}=1$.

**Proof.**  An Appendix A to the proof of Theorem 2. □

We get the following:

$$\varpi_{i}=\sum_{i=1}^{L}\left(\alpha_{i}^{*}-\alpha_{i}\right)\cdot \Phi \left(X_{i}\right),$$

$$b=\frac{1}{L}\sum_{i=1}^{L}\left[Y_{i}-\sum_{i\in RSV}\left(\alpha_{i}^{*}-\alpha_{i}\right)\cdot K\left(X_{i},X_{j}\right)-\frac{\left(L\cdot \sigma_{i}^{2}\cdot \alpha_{i}-C\cdot \lambda_{2}\right)}{C\cdot \lambda_{1}}\right].$$

To estimate ε, we get use the following:

$$\varepsilon =\frac{1}{L}\sum_{j=1}^{L}\left(\sum_{i\in RSV}\left(\alpha_{i}^{*}-\alpha_{i}\right)\cdot K\left(X_{i},X_{j}\right)+b-Y_{j}\right).$$

Thus, the decision function of model GLMH-SVR can be written as follows:

$$f\left(X\right)=\varpi^{T}\cdot \Phi \left(X\right)+b=\sum_{i\in RSV}\left(\alpha_{i}^{*}-\alpha_{i}\right)K\left(X_{i},X\right)+b,$$

where RSVs are samples about $\alpha_{i}^{*}-\alpha_{i}\neq 0$ (called support vectors), parameter vector $\omega \in R^{n}$, $\Phi :R^{n}\to H$, and $K(X_{i},X_{j})$ is the Kernel function.

If the noise in Equation (2) is Gaussian, with zero mean and homoscedasticity, Theorem 1 can be derived by Theorem 2.

## 4. Solution Based on the Augmented Lagrange Multiplier Method

The augmented Lagrange multiplier method (ALM) method [39,40,41] is a class of algorithms for solving equality- and inequality-constrained optimization problems. It solves the dual problem of Equation (7) of model GLM-SVR by applying Newton’s method to a series of constrained problems. By eliminating equality and inequality constraints, the optimization problem of Equation (7) can be reduced to an equivalent unconstrained problem. Gradient descent method or Newton method can be used to solve above problems [24,42,43]. If there are large-scale training samples, some fast optimization techniques can also be combined with the proposed objective function, such as stochastic gradient decent [44].

In this section, we apply Newton’s method to the sequence of inequality and equality constraints and use ALM method to solve model GLM-SVR. Theorem 1 and Theorem 2 provide the algorithms for effectively identifying models GLM-SVR and GLMH-SVR, respectively. The solution based on ALM and algorithm design of model GLM-SVR is given. Similarly, model GLMH-SVR can be solved by the use of ALM.

(1) Let training samples $D_{L}=\left\{\left(X_{1},Y_{1}\right),\left(X_{2},Y_{2}\right),\ldots ,\left(X_{L},Y_{L}\right)\right\}$, where $X_{i}\in R^{n}$, $Y_{i}\in R$, i=1,…,L.

(2) The 10-fold cross-validation strategy is adopted to search the optimal parameters $C,\nu ,\lambda_{1},$ and $\lambda_{2}$ and to select the appropriate kernel function K(•,•).

(3) Solve the optimization problem of Equation (7), and get the optimal solution $\alpha =\left(\alpha_{1},\cdots ,\alpha_{L}\right),\alpha^{*}=\left(\alpha_{1}^{*},\cdots ,\alpha_{L}^{*}\right)$.

(4) Construct the decision function as

$$f\left(X\right)=\varpi^{T}\cdot \Phi \left(X\right)+b=\sum_{i\in RSV}\left(\alpha_{i}^{*}-\alpha_{i}\right)K\left(X_{i},X\right)+b,$$

where $b=\frac{1}{L}\sum_{i=1}^{L}\left[Y_{i}-\sum_{i\in RSV}\left(\alpha_{i}^{*}-\alpha_{i}\right)\cdot K\left(X_{i},X_{j}\right)-\frac{\left(L\cdot \alpha_{i}-C\cdot \lambda_{2}\right)}{C\cdot \lambda_{1}}\right]$

and estimate ε as

$$\varepsilon =\frac{1}{L}\sum_{j=1}^{L}\left(\sum_{i\in RSV}\left(\alpha_{i}^{*}-\alpha_{i}\right)\cdot K\left(X_{i},X_{j}\right)+b-Y_{j}\right).$$

Parameter vector $\varpi \in R^{n}$, $\Phi :R^{n}\to H$ (*H* is Hilbert space), where $\left(\Phi \left(X_{i}\right)\cdot \Phi \left(X_{j}\right)\right)$ is the inner product of *H* and $K\left(X_{i},X_{j}\right)=\left(\Phi \left(X_{i}\right)\cdot \Phi \left(X_{j}\right)\right)$ is the Kernel function.

## 5. Case Study

In this section, a case study is implemented to demonstrate the effectiveness of the proposed model GLM-SVR through comparisons with other techniques for training-set $D_{f}$ from Heilongjiang, China. This case study includes three subsections: Data collection and analysis in Section 5.1; evaluation criteria for forecasting performance in Section 5.2; and short-term wind speed forecasting of a real dataset in Section 5.3.

### 5.1. Analysis of Wind Speed Mixture Noise Characteristics

**Figure 2:**
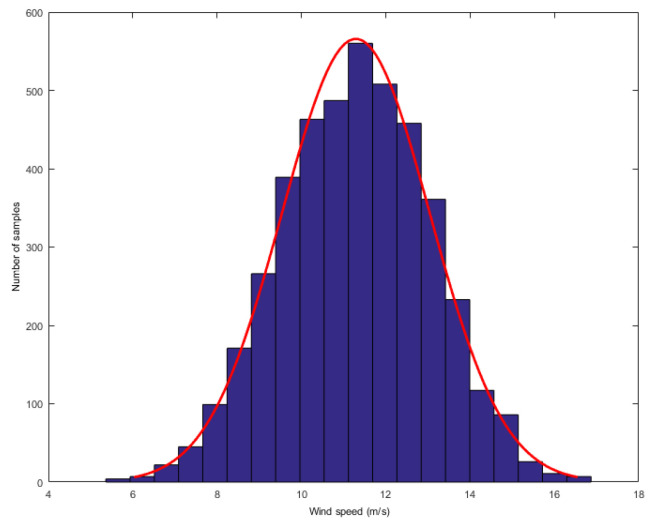
The distribution of high wind speed.

**Figure 3:**
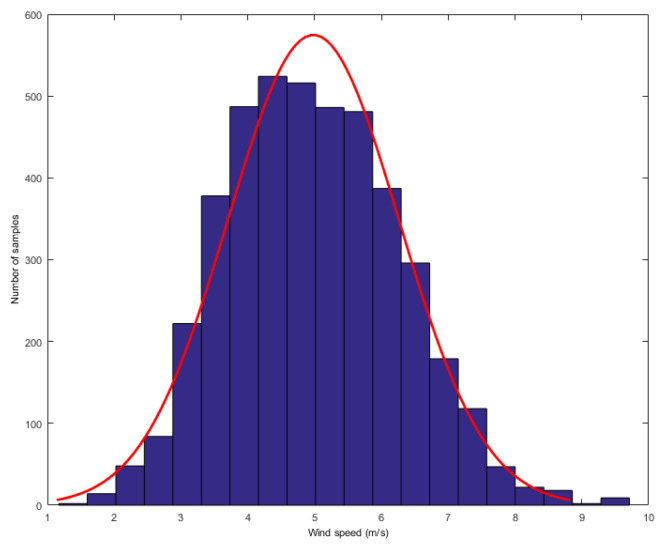
The distribution of low wind speed.

In order to analyze the mixture noise characteristics of wind speed forecasting error, we collected wind speed dataset from Heilongjiang, China. The dataset consists of one-year wind speed data, recording the wind speed values every 10 min. We first found the Gauss-Laplace mixture noise in the above data. The researchers have found that turbulence is the major cause of the wind speed’s strong random fluctuation uncertainty. From wind energy perspective, the most striking characteristic of the wind resource is its variability. Now we display the distributions of wind speed. We obtain a value for wind speed after every 10 min and compute the histograms of wind speed in one or two hours. Two typical distributions are given as follows: one was computed when the wind speed was higher and the other was computed when the wind speed was lower, as shown in Figure 2 and Figure 3, respectively.

**Figure 4:**
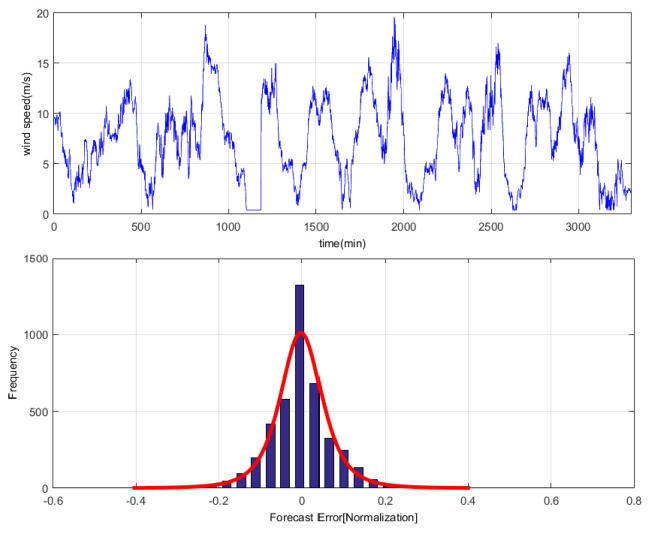
Gauss-Laplace mixture distribution of wind speed prediction error.

To analyze one-month time series of wind speed dataset, the persistence method is used to investigate the distribution of wind speed prediction errors [28]. The result indicates that the error ξ does not satisfy the single distribution but approximately obeys the Gauss-Laplace mixed distribution and that the PDF of ξ is $P\left(\xi \right)=\frac{1}{2}e^{-|\xi |}\cdot \frac{1}{2\sigma^{2}}\xi^{2}$, as shown in Figure 4. This is a regression learning task of mixture noise.

### 5.2. Evaluation Criteria for Forecasting Performance

As we all know, no prediction model forecasts perfectly. There are also certain criteria, such as mean absolute error (MAE), the root mean square error (RMSE), mean absolute percentage error (MAPE), and standard error of prediction (SEP), which are used to evaluate the predictive performance of models ν-SVR, GN-SVR, and GLM-SVR. The four criteria are defined as follows:

(10) $$MAE=\frac{1}{L}\sum_{i=1}^{L}\left|Y_{i}^{\prime }-Y_{i}\right|,$$

(11) $$MAPE=\frac{1}{L}\sum_{i=1}^{L}\frac{|Y_{i}^{\prime }-Y_{i}|}{Y_{i}}\times 100\%,$$

(12) $$RMSE=\sqrt{\frac{1}{L}\sum_{i=1}^{L}{\left(Y_{i}^{\prime }-Y_{i}\right)}^{2}},$$

(13) $$SEP=\frac{RMSE}{\bar{Y}}\times 100\%,$$

Among them, *L* is the size of the training samples, $Y_{i}$ is the *i*th actual measured data, $Y_{i}^{\prime }$ is the *i*th forecasted result, and $\bar{Y}$ is the mean value of observations of all selected samples in the training-set $D_{L}$ [45,46,47]. The MAE reveals how similar the predicted values are to the observed values, whereas the RMSE measures the overall deviation between the predicted and observed values. MAPE is the ratio between errors and observed values, and SEP is the ratio between RMSE and mean values of observations. The indicators MAPE and SEP are unit-free measures of accuracy for predicting wind series and are sensitive to small changes.

### 5.3. Short-Term Wind Speed Prediction of Real Dataset

In this subsection, we demonstrate the validity of the proposed model by conducting experiments on wind speed dataset from Heilongjiang Province, China. The data records more than one year of wind speeds. The average wind speed in 10 min are stored. As a whole, 62,466 samples with 4 attributes: mean, variance, minimum, and maximum. We first extracted 2160 consecutive data points (from 1 to 2160; the time length is 15 days) as the training set and 720 consecutive data points (from 2161 to 2880, the time length is 5 days) as the testing set. We transform the original sequence into a multivariate regression task using mode $\vec{X_{i}}$ = $\left(X_{i-10},X_{i-9},\cdots ,X_{i-1},X_{i}\right)$ as an input vector to predict $X_{i+step}$, in which the vector orders of wind speed is determined by the chaotic operator network method [48], where $X_{j}$ is the real value of wind speed at time j(j=i−10,i−9,⋯,i), step=1,3,5, that is to say, using the above mode to predict the wind speed at each point $X_{i}$ after 10-min, 30-min, and 50-min, respectively.

Models ν-SVR, GN-SVR, and GLM-SVR have been implemented in Matlab 7.8 programming language. The initial parameters are C∈[1,201], ν∈(0,1], $\lambda_{1},$ and $\lambda_{2}\in \left[0,1\right]$. We use the 10-fold cross validation strategy to find optimal positive parameters $C,\nu ,\lambda_{1},$ and $\lambda_{2}$, of which the parameters selection technology is studied in detail in References [49,50]. In this article, the parameter assignments are as follows: $C=181,\nu =0.5,\lambda_{1}=0.5,\;\mathrm{and}\;\lambda_{2}=0.5$. Many practical applications display that polynomial and Gaussian kernels perform well under general smooth assumptions. In this case study, polynomial and Gaussian kernel functions are utilized in models ν-SVR, the ν-SVR model of Gauss homoscedastic noise (GN-SVR), and GLM-SVR as below [51,52].

$$K\left(X_{i},X_{j}\right)={\left(\left(X_{i},X_{j}\right)+1\right)}^{d}$$

and

$$K\left(X_{i},X_{j}\right)=e^{-\frac{∥X_{i}-X_{j}{∥}^{2}}{\sigma^{2}}},$$

where *d* is a positive integer and σ is positive.

**Figure 5:**
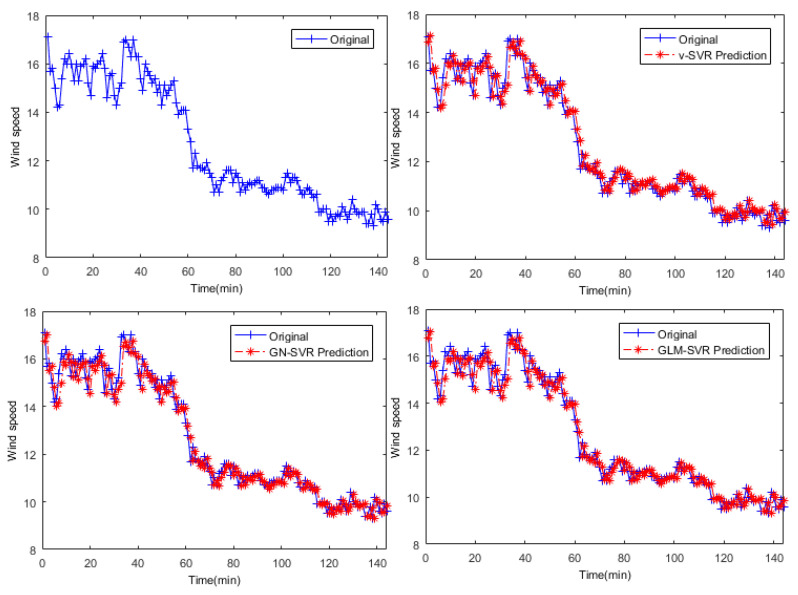
Result of wind speed prediction at $X_{i}$-point after 10 min.

**Figure 6:**
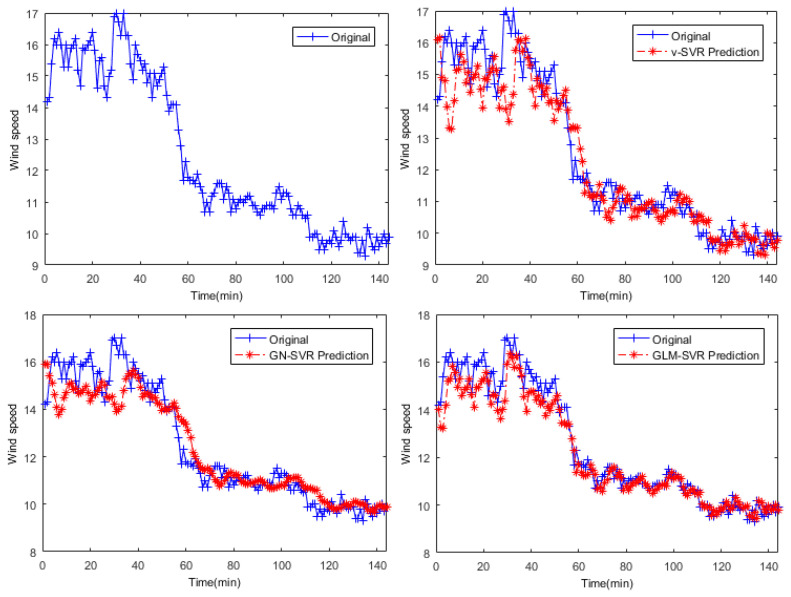
Result of wind speed prediction at $X_{i}$-point after 30 min.

**Figure 7:**
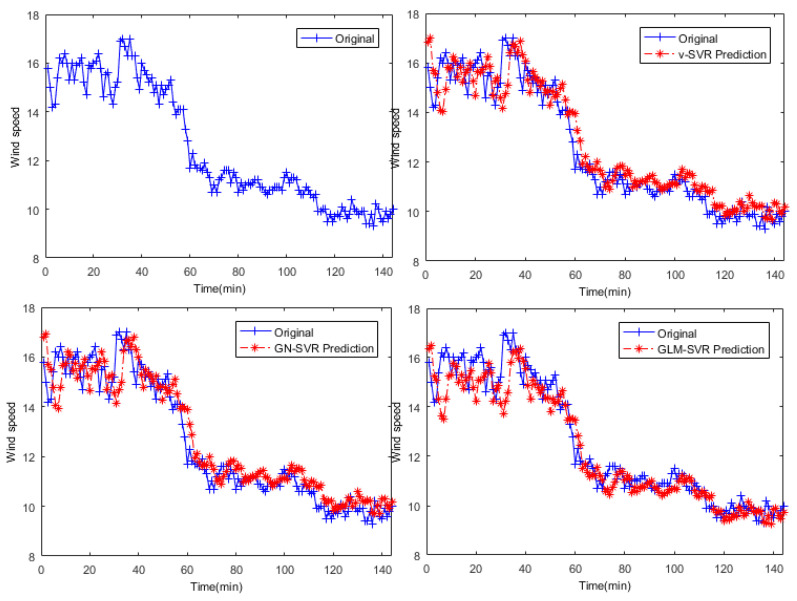
Result of wind speed prediction at $X_{i}$-point after 50 min.

In Figure 5, Figure 6 and Figure 7, the results of wind speed prediction at the $X_{i}$ point for models ν-SVR, GN-SVR, and GLM-SVR are obtained after 10 min, 30 min, and 50 min, respectively.

**Figure 8:**
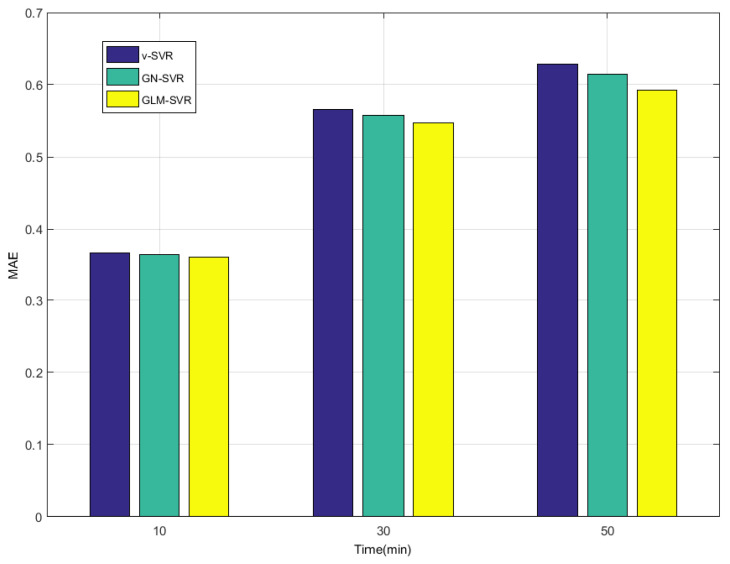
Error statistical histograms of index MAE for wind speed prediction at $X_{i}$-point.

**Figure 9:**
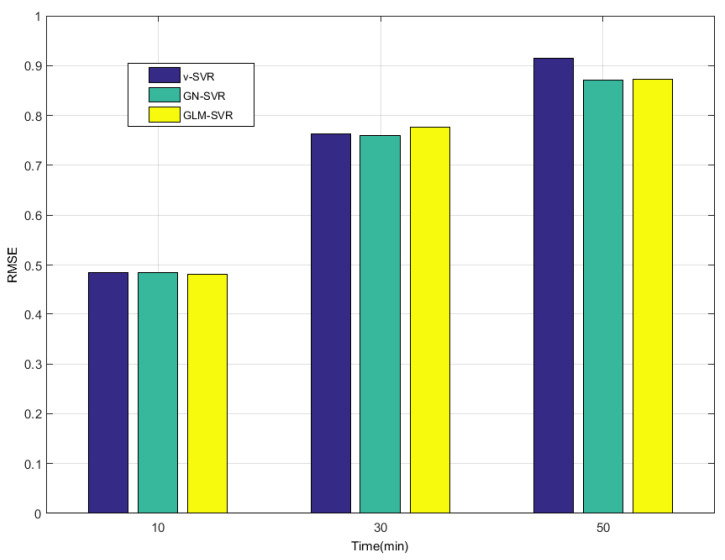
Error statistical histograms of index RMSE for wind speed prediction at $X_{i}$-point.

**Figure 10:**
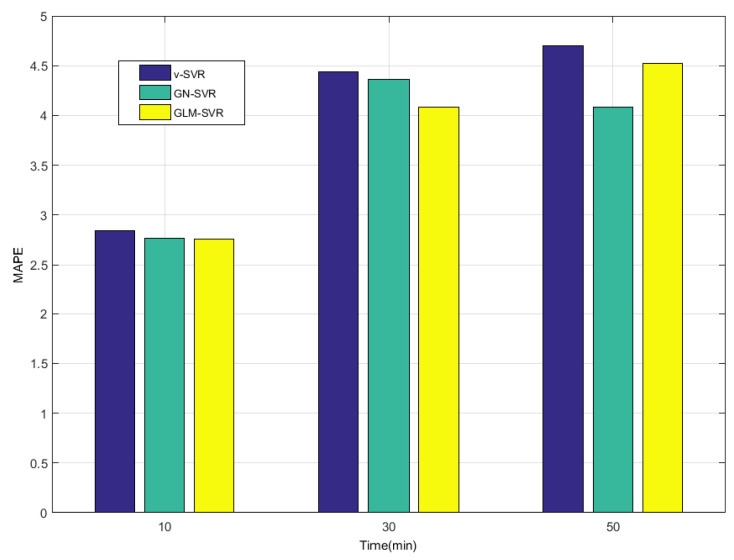
Error statistical histograms of index MAPE for wind speed prediction at $X_{i}$-point.

**Figure 11:**
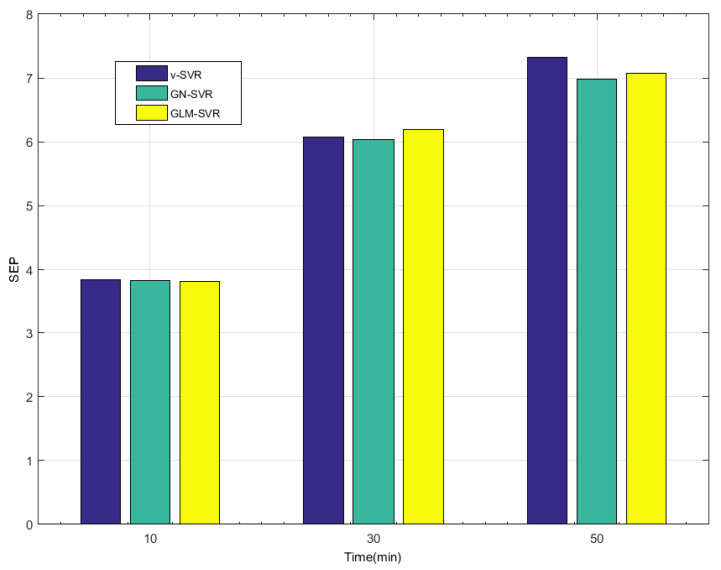
Error statistical histograms of index SEP for wind speed prediction at $X_{i}$-point.

In Table 1, Table 2 and Table 3 and Figure 8, Figure 9, Figure 10 and Figure 11, indicators MAE,MAPE,RMSE, and SEP of wind speed prediction at $X_{i}$-point for models ν-SVR, GN-SVR, and GLM-SVR are obtained after 10 min, 30 min, and 50 min, respectively.

From Table 1, Table 2 and Table 3 and Figure 5, Figure 6, Figure 7, Figure 8, Figure 9, Figure 10 and Figure 11, in most cases, it can be concluded that the error calculation of model GLM-SVR is better than that of models ν-SVR and GN-SVR. As the prediction horizon increases to 30 min and 50 min, the errors obtained by different models rise and the relative difference decreases. However, as can be seen from Table 1, Table 2 and Table 3, the Gauss-Laplace mixture noise model is slightly superior to the classical model in terms of all indicators: MAE, MAPE, RMSE, and SEP.

## 6. Conclusions

The noise distribution is complex or unknown in the real world; it is almost impossible for a single distribution to describe real noise. This article describes the main results: (1) optimal empirical risk loss for mixture noise model is derived by the Bayesian principle; (2) model ν-SVR of the Gauss-Laplace mixture homoscedastic noise (GLM-SVR) and Gauss-Laplace mixture heteroscedastic noise (GLMH-SVR) for complex or unknown noise is developed; (3) the dual problems of GLM-SVR and GLMH-SVR are derived by introducing Lagrange functional $L_{\varpi }$; (4) the ALM method is applied to solve model GLM-SVR, which guarantees the stability and validity; and (5) model GLM-SVR is applied to short-term wind speed forecasting using historical data to predict future wind speed at a certain time. The experimental results on real-world data of wind speed confirm the effectiveness of the proposed technique.

Analogously, we can study the Gauss-Laplace mixture noise model of classification, which will be successfully used to solve the classification problem for complex or unknown noise characteristics.

## Figures and Tables

**Table 1:** Error statistic of wind speed prediction at $X_{i}$-point after 10 min.

Model	MAE (m/s)	RMSE (m/s)	MAPE (%)	SEP (%)
ν-SVR	0.3671	0.4854	2.84	3.84
GN-SVR	0.3641	0.4845	2.77	3.83
GLM-SVR	0.3616	0.4813	2.76	3.81

**Table 2:** Error statistic of wind speed prediction at $X_{i}$-point after 30 min.

Model	MAE (m/s)	RMSE (m/s)	MAPE (%)	SEP (%)
ν-SVR	0.5655	0.7631	4.44	6.08
GN-SVR	0.5577	0.7589	4.36	6.04
GLM-SVR	0.5468	0.7773	4.08	6.19

**Table 3:** Error statistic of wind speed prediction at $X_{i}$-point after 50 min.

Model	MAE (m/s)	RMSE (m/s)	MAPE (%)	SEP (%)
ν-SVR	0.6281	0.9142	4.70	7.32
GN-SVR	0.6146	0.8711	4.61	6.98
GLM-SVR	0.5920	0.8734	4.52	7.07

## Abbreviations

The following abbreviations are used in this manuscript:

SVR	Support vector regression
GLM-SVR	SVR model of Gauss-Laplace mixture heteroscedastic noise
GLMH-SVR	SVR model of Gauss-Laplace mixture homoscedastic noise
GN-SVR	SVR model of Gauss homoscedastic noise
ALM	augmented Lagrange multiplier method

## Appendix A

**Proof** **of** **Theorem** **2.** Let’s take Lagrange functional $L_{\varpi }\left(\varpi ,b,\alpha ,\alpha^{*},\xi ,\xi^{*},\eta ,\eta^{*},\varepsilon \right)$ as:

$$\begin{array}{c} L_{\varpi }=\frac{1}{2}\varpi^{T}\cdot \varpi +\frac{C}{L}\cdot \left[\nu \cdot \varepsilon +\frac{\lambda_{1}}{2}\cdot \sum_{i=1}^{L}\frac{1}{\sigma_{i}^{2}}\cdot \xi_{i}^{2}+\frac{1}{{\left(\sigma_{i}^{*}\right)}^{2}}\cdot {\left(\xi_{i}^{*}\right)}^{2}\right)\\ +\lambda_{2}\cdot \sum_{i=1}^{L}\left(\xi_{i}+\xi_{i}^{*}\right)]-\gamma \varepsilon -\sum_{i=1}^{L}\left(\eta_{i}\xi_{i}+\eta_{i}^{*}\xi_{i}^{*}\right)\\ -\sum_{i=1}^{L}\alpha_{i}\left(\xi_{i}+y_{i}-\varpi^{T}\cdot \Phi \left(x_{i}\right)-b+\varepsilon \right)\\ -\sum_{i=1}^{L}\alpha_{i}^{*}\left(\xi_{i}^{*}-y_{i}+\varpi^{T}\cdot \Phi \left(x_{i}\right)+b+\varepsilon \right). \end{array}$$

To minimize $L_{\varpi }$, let’s find partial derivative $\varpi ,b,\xi ,\xi^{*},\varepsilon$, respectively. On the basis of KKT(Karush-Kuhn-Tucker) conditions, get

$$\nabla_{\varpi }\left(L\right)=0,\nabla_{b}\left(L\right)=0,\nabla_{\varepsilon }\left(L\right)=0,\nabla_{\xi }\left(L\right)=0,\nabla_{\xi^{*}}\left(L\right)=0.$$

And have

$$\varpi_{i}=\sum_{i=1}^{L}\left(\alpha_{i}^{*}-\alpha_{i}\right)\cdot \Phi \left(x_{i}\right),\sum_{i=1}^{L}\left(\alpha_{i}^{*}-\alpha_{i}\right)=0,$$

$$\frac{C}{L}\cdot \nu -\gamma -\sum_{i=1}^{L}\left(\alpha_{i}^{*}+\alpha_{i}\right)=0,$$

$$\frac{C}{L}\cdot \left(\frac{\lambda_{1}\cdot \xi_{i}}{\sigma_{i}^{2}}+\lambda_{2}\right)-\eta_{i}-\alpha_{i}=0,$$

$$\frac{C}{L}\cdot \left(\frac{\lambda_{1}\cdot \xi_{i}^{*}}{{\left(\sigma_{i}^{*}\right)}^{2}}+\lambda_{2}\right)-\eta_{i}^{*}-\alpha_{i}^{*}=0,$$

Substituting extreme conditions into $L_{\varpi }$ and seeking maximum of $\alpha ,\alpha^{*}$, Dual Problem (9) of Primal Problem (8) be derived. □

## References

[1] European Wind Energy Association, Wind Force 12. http://www.ewea.org/doc/WindForce12.

[2] Sfetsos A. (2008). A comparison of various forecasting techniques applied to mean hourly wind speed time series. Renew. Energy.

[3] Calif R., Schmitt F. (2012). Modeling of atmospheric wind speed sequence using a lognormal continuous stochastic equation. J. Wind Eng. Inst. Aerodyn..

[4] Calif R., Schmitt F. (2014). Multiscaling and joint multiscaling of the atmospheric wind speed and the aggregate power output from a wind farm. Nonlinear Process. Geophys..

[5] Jung J., Broadwater R.P. (2014). Current status and future advances for wind speed and power forecasting. Renew. Sustain. Energy Rev..

[6] Zhang C., Wei H., Zhao J., Liu T., Zhu T., Zhang K. (2016). Short-term wind speed forecasting using empirical mode decomposition and feature selection. Renew. Energy.

[7] Wang Y., Hu Q.H., Li L.H., Foley A.M., Srinivasan D. (2019). Approaches to wind power curve modeling: A review and discussion. Renew. Sustain. Energy Rev..

[8] Wang J.Z., Zhang N., Lu H.Y. (2019). A novel system based on neural networks with linear combination framework for wind speed forecasting. Energy Convers. Manag..

[9] Sun L., Liu R.N., Xu J.C., Zhang S.G. (2019). An adaptive density peaks clustering method with Fisher linear discriminant. IEEE Access..

[10] Sun L., Wang L.Y., Qian Y.H., Xu J.C., Zhang S.G. (2019). Feature selection using Lebesgue and entropy measures for incomplete neighborhood decision systems. Knowl.-Based Syst..

[11] Sun L., Xu J.C., Liu S.W., Zhang S.G., Li Y., Shen C.A. (2018). A robust image watermarking scheme using Arnold transform and BP neural network. Neural Comput. Appl..

[12] Liu Z.T., Li C.G. (2015). Censored regression with noisy input. IEEE Trans. Signal Process..

[13] Sun L., Zhang X.Y., Qian Y.H., Xu J.C., Zhang S.G., Tian Y. (2019). Joint neighborhood entropy-based gene selection method with fisher score for tumor classification. Appl. Intell..

[14] Wu Q. (2010). A hybrid-forecasting model based on Gaussian support vector machine and chaotic particle swarm optimization. Expert Syst. Appl..

[15] Wu Q., Law R. (2011). The forecasting model based on modified SVRM and PSO penalizing gaussian noise. Expert Syst. Appl..

[16] Meng D.Y., Torre F.D.L. Robust matrix factorization with unknown noise. Proceedings of the IEEE International Conference on Computer Vision (ICCV 2013).

[17] Hu Q.H., Zhang S.G., Xie Z.X., Mi J.S., Wan J. (2014). Noise model based *ν*-Support vector regression with its application to short-term wind speed forecasting. Neural Netw..

[18] Zhang S.G., Hu Q.H., Xie Z.X., Mi J.S. (2015). Kernel ridge regression for general noise model with its application. Neurocomputing.

[19] Hu Q.H., Zhang S.G., Yu M., Xie Z.X. (2016). Short-term wind speed or power forecasting with heteroscedastic support vector regression. IEEE Trans. Sustain. Energy.

[20] Ma C.F. (2010). Optimization Method and the Matlab Programing Design.

[21] Schölkopf B., Smola A.J., Williamson R.C., Bartlett P.L. (2000). New support vector algorithms. Neural Comput..

[22] Suykens J.A.K., Lukas L., Vandewalle J. Sparse approximation using least square vector machines. Proceedings of the IEEE International Symposium on Circuits and Systems.

[23] Suykens J.A.K., Van Gestel T., Brabanter J.D., Moor B.D., Vandewalle J. (2002). Least Squares Support Vector Machines.

[24] Pontil M., Mukherjee S., Girosi F. (2000). On the Noise Model of Support Vector Machines Regression.

[25] Bofinger S., Luig A., Beyer H.G. Qualification of wind power forecasts. Proceedings of the Global Wind Power Conference (GWPC 2002).

[26] Zhang Y., Wan Q., Zhao H.P., Yang W.L. (2007). Support vector regression for basis selection in Laplacian noise environment. IEEE Signal Lett..

[27] Randazzo A., Abou-Khousa M.A., Pastorino M., Zoughi R. (2007). Direction of arrival estimation based on support Vector regression: Experimental Validation and Comparison with Music. IEEE Antennas Wirel. Propag. Lett..

[28] Bludszuweit H., Antonio J., Llombart A. (2008). Statistical analysis of wind power forecast error. IEEE Trans. Power Syst..

[29] Jiang P., Li P.Z. (2017). Research and Application of a New Hybrid Wind Speed Forecasting Model on BSO algorithm. J. Energy Eng..

[30] Bishop C.M. (2006). Pattern Recognition and Machine Learning.

[31] Du P., Wang J.Z., Guo Z.H., Yang W.D. (2017). Research and application of a novel hybrid forecasting system based on multi-objective optimization for wind speed forecasting. Energy Convers. Manag..

[32] Jiang Y., Huang G.Q. (2016). A hybrid method based on singular spectrum analysis, firefly algorithm, and BP neural network for short-term wind speed forecasting. Energies.

[33] Jiang Y., Huang G.Q. (2017). Short-term wind speed prediction: Hybrid of ensemble empirical mode decomposition, feature selection and error correction. Energy Convers. Manag..

[34] Wang H.B., Wang Y., Hu Q.H. (2017). Self-adaptive robust nonlinear regression for unknown noise via mixture of Gaussians. Neurocomputing.

[35] Shevade S.K., Keerthi S.S., Bhattacharyya C., Murthy K.R.K. (2000). Improvements to the SMO Algorithm for SVM Regression. IEEE Trans. Neural Netw..

[36] Chu W., Keerthi S.S., Ong C.J. (2004). Bayesian support vector regression using a unified loss function. IEEE Trans. Neural Netw..

[37] Klaus-Robert Sebastia M.M. (2001). An introduction to kernel-based learning algorithms. IEEE Trans. Neural Netw..

[38] Sun L., Liu R.N., Xu J.C., Zhang S.G., Tian Y. (2018). An affinity propagation clustering method using hybrid kernel function with LLE. IEEE Access.

[39] Rockafellar R.T. (1973). The multiplier method of Hestenes and Powell applied to convex programming. J. Optim. Theory Appl..

[40] Rockafellar R.T. (1974). Augmented Lagrange Multiplier Functions and Duality in Nonconvex Programming. SIAM J. Control.

[41] Sun L., Chen S.S., Xu J.C., Tian Y. (2019). Improved Monarch Butterfly Optimization algorithm based on opposition-based learning and random local perturbation. Complexity.

[42] Boyd S., Vandenberghe L. (2004). Convex Optimization.

[43] Wang S.X., Zhang N., Wu L., Wang Y.M. (2016). Wind speed forecasting based on the hybrid ensemble empirical mode decomposition and GA-BP neural network method. Renew. Energy.

[44] Léon B. Large-Scale Machine Learning with Stochastic Gradient Descent. Proceedings of the 19th International Conference on Computational Statistics (COMPSTAT’2010).

[45] Fabbri A., Román T.G.S., Abbad J.R., Quezada V.H.M. (2005). Assessment of the cost associated with wind generation prediction errors in a liberalized electricity market. IEEE Trans. Power Syst..

[46] Guo Z.H., Zhao J., Zhang W.Y., Wang J.Z. (2011). A corrected hybrid approach for wind speed prediction in Hexi Corridor of China. Energy.

[47] Wang J.Z., Hu J.M. (2015). A robust combination approach for short-term wind speed forecasting and analysis-Combination of the ARIMA, ELM, SVM and LSSVM forecasts using a GPR model. Energy.

[48] Xiu C.B., Guo F.H. (2013). Wind speed prediction by chaotic operator network based on Kalman Filter. Sci. China Technol. Sci..

[49] Abdoos A.A. (2016). A new intelligent method based on combination of VMD and ELM for short term wind power forecasting. Neurocomputing.

[50] Chalimourda A., Schölkopf B., Smola A.J. (2004). Experimentally optimal *ν* in support vector regression for different noise models and parameter settings. Neural Netw..

[51] Cherkassky V., Ma Y. (2004). Practical selection of SVM parameters and noise estimation for SVM regression. Neural Netw..

[52] Sun L., Zhang X.Y., Qian Y.H., Xu J.C., Zhang S.G. (2019). Feature selection using neighborhood entropy-based uncertainty measures for gene expression data classification. Inf. Sci..

